# The effects of patchouli alcohol and combination with cisplatin on proliferation, apoptosis and migration in B16F10 melanoma cells

**DOI:** 10.1111/jcmm.17745

**Published:** 2023-04-10

**Authors:** Kai‐Fu Chang, Hung‐Chih Lai, Shan‐Chih Lee, Xiao‐Fan Huang, Ya‐Chih Huang, Tien‐Erh Chou, Chih‐Yen Hsiao, Nu‐Man Tsai

**Affiliations:** ^1^ Department of Medical Laboratory and Biotechnology Chung Shan Medical University Taichung Taiwan, R.O.C; ^2^ Division of Hematology and Oncology, Department of Internal Medicine Shin‐Kong Wu Ho‐Su Memorial Hospital Taipei Taiwan, R.O.C; ^3^ Institute of Pharmacology National Taiwan University Taipei Taiwan, R.O.C; ^4^ Department of Medical Imaging and Radiological Sciences Chung Shan Medical University Taichung Taiwan, R.O.C; ^5^ Department of Medical Imaging Chung Shan Medical University Hospital Taichung Taiwan, R.O.C; ^6^ Institute of Medicine Chung Shan Medical University Taichung Taiwan, R.O.C; ^7^ Division of Nephrology, Department of Internal Medicine Ditmanson Medical Foundation Chia‐Yi Christian Hospital Chia‐Yi Taiwan, R.O.C; ^8^ Clinical Laboratory Chung Shan Medical University Hospital Taichung Taiwan, R.O.C; ^9^ Department of Life‐and‐Death Studies Nanhua University Chiayi Taiwan, R.O.C

**Keywords:** cell apoptosis, drug combination, melanoma, migration, patchouli alcohol

## Abstract

Melanoma is a highly metastatic cancer with a low incidence rate, but a high mortality rate. Patchouli alcohol (PA), a tricyclic sesquiterpene, is considered the main active component in Pogostemon cablin Benth, which improves wound healing and has anti‐tumorigenic activity. However, the pharmacological action of PA on anti‐melanoma remains unclear. Thus, the present study aimed to investigate the role of PA in the proliferation, cell cycle, apoptosis and migration of melanoma cells. These results indicated that PA selectively inhibited the proliferation of B16F10 cells in a dose‐ and time‐dependent manner. It induced cell cycle arrest at the G_0_/G_1_ phase and typical morphological changes in apoptosis, such as chromatin condensation, DNA fragmentation and apoptotic bodies. In addition, PA reduced the migratory ability of B16F10 cells by upregulating E‐cadherin and downregulating p‐Smad2/3, vimentin, MMP‐2 and MMP‐9 expression. PA was also found to strongly suppress tumour growth in vivo. Furthermore, PA combined with cisplatin synergistically inhibited colony formation and migration of B16F10 cells and attenuated the development of resistance to treatment. Therefore, the results of this study indicate that PA may play a pivotal role in inducing apoptosis and reducing the migration of melanoma cells, and may thus be a potential candidate for melanoma treatment.

## 1 BACKGROUND

The latest report from the World Health Organization in 2018 showed that there were 288,000 new cases and 61,000 deaths associated with melanoma.^1^ Melanoma is a highly malignant cancer with a high mortality rate mainly because of its high metastatic ability and resistance to various treatments.^2^ When patients are diagnosed with melanoma, most have advanced to middle or advanced stages, with obvious symptoms of metastasis. Once the cancerous cells spread to other tissues or organs of the body, they cannot be cured by surgery, which is related to more than 90% of deaths by melanoma.^3^ Cytotoxic chemotherapy, including a combination of cisplatin (CDDP) and doxorubicin (DOXO), has been widely used as the main treatment for melanoma.^4^ However, it induces apoptosis in both melanoma and normal tissues and exhibits undesirable side effects, such as bone marrow toxicity, nephrotoxicity, hepatotoxicity and gastrointestinal toxicity.^5^, ^6^ Although chemotherapy suppresses the growth of tumours, this effect is not long‐lasting in most cases, resulting in drug resistance in melanoma, leading to treatment failure.^7^ Together, the clinical benefits of these approaches are affected by the cytotoxicity, drug resistance and drug selection of cells. Hence, it is important to determine how to prolong the effective duration of drugs and reduce side effects to improve the quality of life of patients. Conventional drugs are insufficient to improve the status of treatment, and it is necessary to develop new chemotherapeutic drugs to overcome the current limitations of melanoma therapy.

The demand for the development of novel anticancer drugs has been directed towards research on potentially useful natural products in herbs, vegetables and fruits.^8^, ^9^ Approximately 40% of approved commercial drugs and 64.9% of existing anti‐cancer drugs are derived from natural products or their derivatives.^10^ They are an important source of compounds with biological activities against various diseases and have low toxicity and side effects.^11^ In addition, there are several studies that used herbal medicine to treat malignant melanoma, which indicate anti‐proliferative, pro‐apoptotic and anti‐metastatic effects. For example, hispidulin, a flavone distributed in plants of the *Asteraceae*, reduced cell growth by decreasing AKT and ERK phosphorylation, inducing apoptosis via activation of caspases and inhibiting cell migration through downregulation of matrix metalloproteinase‐2 expression in A2058 human melanoma cells.^12^ Theaflavin, a primary pigment of tea, suppressed cell proliferation, induced early and late apoptosis and inhibited migration/invasion through the regulation of relative mRNA and protein expression in B16F10 mouse melanoma cells.^13^

*Pogostemon cablin* Benth. is a medicinal plant that is commonly used to treat fever, headache, nausea, diarrhoea and facial diseases and is widely cultivated in the Philippines, Malaysia, India and China.^14^, ^15^, ^16^ The extracts of P. *cablin* are one of the most important ingredients in cosmetics and exhibit soothing and anti‐dermatophytic, antioxidative and anti‐inflammatory effects on the skin.^16^, ^17^, ^18^ Patchouli alcohol (PA), a tricyclic sesquiterpene, is considered a major effective component of P. *cablin* and is used in the quality control of patchouli oil in the pharmaceutical industry. Several studies have demonstrated that PA has multiple effects, such as anti‐oxidation,^19^ anti‐inflammatory,^20^ anti‐influenza virus,^21^ prevention of UV‐induced skin photoaging,^22^ improvement of wound healing,^23^ protection against myocardial ischaemia‐reperfusion injury^24^ and anti‐tumorigenic activity in colorectal, lung and prostate cancer.^25^, ^26^, ^27^ However, despite these diverse beneficial effects, studies on the use of PA in the treatment of melanoma are lacking. The present study aimed to investigate the anticancer effects of PA on melanoma cells. The current data revealed that PA suppresses the growth of B16F10 cells both in vitro and in vivo by regulating proliferation, apoptosis and migration. These findings indicate that PA may be a potential compound for the treatment of melanoma and provide more insights for the development of novel agents or functional foods from medicinal plants.

## 2 MATERIALS AND METHODS

### 2.1 Chemicals and reagents

Patchouli alcohol and cisplatin were purchased from Wuhan ChemFaces Biochemical Co., Ltd. and the Cayman Chemical Company, respectively. 3‐(4,5‐dimethylthiazol‐2‐yl)‐2,5‐diphenyltetrazolium bromide (MTT), dimethyl sulfoxide (DMSO) and propidium iodide (PI) were purchased from Sigma‐Aldrich and terminal deoxynucleotidyl transferase dUTP nick‐end labelling (TUNEL) assay kit was purchased from Roche. Supersensitive link‐label immunohistochemistry (IHC) detection system and 3,3′‐diaminobenzidine (DAB) were purchased from BioGenex. For western blotting and IHC staining experiments, primary antibodies against proliferating cell nuclear antigen (PCNA), cyclin D1, cyclin‐dependent kinase 4 (CDK4), caspase‐8, caspase‐9, caspase‐3, vascular endothelial growth factor (VEGF), matrix metalloproteinase (MMP)‐2 and MMP‐9 were purchased from Santa Cruz Biotechnology; Smad2/3, p‐Smad2/3 were purchased from Cell Signalling Technology; E‐cadherin, vimentin and β‐actin were purchased from iReal Biotechnology Co., Ltd. In vitro experiments, PA (purity ≥98%) was prepared as a 224.9 mM stock solution in DMSO and stored at −20°C. The stock solution was diluted with the complete medium for subsequent applications, containing <1% final concentration of DMSO. In vivo experiments, the powder of PA was dissolved in mineral oil and prepared as 75 and 150 mg/kg for subcutaneous injection. An equal volume of DMSO or mineral oil was used as a negative control in vitro and in vivo experiments, respectively.

### 2.2 Cell culture

The murine B16F10 melanoma and NIH/3T3 fibroblast cell lines were purchased from American Type Culture Collection (ATCC) and cultured in Dulbecco's Modified Eagle's Medium (DMEM) containing 10% fetal bovine serum (FBS), HEPES buffer solution, sodium pyruvate and penicillin/streptomycin at 37°C in a humidified 5% CO_2_ incubator. All the reagents used for cell culture were purchased from Gibco.

### 2.3 Cell viability assay

The inhibitory effect of PA on B16F10 cells was evaluated via cell viability using the MTT assay. The cells were seeded into 96‐well plates at 5 × 10^3^ cells/well in 100 μL medium for 24 h adherence (40%–50% confluence), followed by treatment with PA (0, 28.1, 56.2, 112.4 and 224.9 μM) or cisplatin (0, 4.2, 8.3, 16.6 and 33.2 μM) for 24, 48 and 72 h. Then, MTT solution (500 μg/mL) in the medium was added to each well and incubated at 37°C for 4 h. Optical density (OD) was measured at 550 nm using a microplate reader (SpectraMax Plus 384, Molecular Devices). Inhibitory rate (%) = [1 − (PA − treated OD/control OD)] × 100%. The 50% inhibitory concentration (IC_50_) was calculated using regression analysis. The experiments were repeated at least three times.

In drug combination analysis, cells were plated in 96‐well culture plates (5 × 10^3^ cells/well) for 24 h, and treated with PA (0, 22.5, 45.0, 67.5 and 89.9 μM) combined with cisplatin (0, 3.3, 6.6 and 13.3 μM) for 48 h, and detected the cell viability using an MTT assay. The drug–drug interactions were determined using combination index (CI) to evaluate the occurrence of synergism (CI < 1), additive effect (CI = 1) and antagonism (CI > 1), calculated by CompuSyn software (ComboSyn Inc.) based on the Chou‐Talalay method.

Detecting inhibitory effects according to wound‐healing condition, B16F10 cells were harvested and 1.2 × 10^4^ cells were seeded in a 96‐well plate and incubated for 24 h. When the cells reached 90% confluence, the cultured medium was replaced with the medium containing 1% FBS. Cells were treated with PA (0, 22.5, 45 and 67.5 μM) for 24 and 48 h, and measured the cell viability using an MTT assay.

### 2.4 Cell cycle analysis

The cell cycle distribution in PA‐treated cells was determined by PI staining. Cells were treated with 89.9 μM PA for 0, 6, 12, 24 and 48 h. Then, the treated cells were harvested and incubated with the solution of PI (40 μg/mL) and RNase A (100 μg/mL) overnight at 4°C. The cell cycle distribution was detected by FACScan flow cytometry (Beckton Dickinson) and calculated using FlowJo software (Treestar).

### 2.5 Apoptosis detection

DNA cleavage was assessed using the TUNEL assay kit following the manufacturer's instructions. B16F10 cells were plated in 10 cm dishes (2 × 10^6^ cells/dish), cultured for 24 h and then treated with 89.9 μM PA. After further 6–48 h, the PA‐treated cells were fixed with 10% neutral buffered formalin for 10 min and stored at 4°C. Treated cells or tumour tissues were incubated with 3% H_2_O_2_ in methanol, permeabilized with 0.1% Triton X‐100 in 0.1% sodium citrate on ice and incubated with TUNEL solution for 1 h at 37°C. PI staining (red fluorescence) was used as a contrast dye to highlight the nuclei. TUNEL assay results (green fluorescence) were observed using a Zeiss Axioskop 2 plus microscope (Carl Zeiss, Thornwood, NY, USA).

### 2.6 Western blot analysis

Treated cells were lysed in ice‐cold radioimmunoprecipitation assay (RIPA) buffer (Bio Basic Inc.) containing a protease inhibitor cocktail (Amresco Inc.) and phosphatase inhibitor cocktail (Bionovas) for 30 min on ice. Protein lysates were centrifuged at 14,000*g* for 30 min at 4°C to collect the lysate supernatant, and protein concentrations were measured using a BCA Protein Assay Kit (Pierce; Thermo Scientific). Protein lysates (20 μg per lane) were resolved by electrophoresis on 8%–12.5% sodium dodecyl sulfate (SDS) polyacrylamide gels and transferred onto 0.22 μm polyvinylidene difluoride (PVDF) membranes (FluoroTrans, PALL). The membranes were blocked with 5% skim milk and incubated with primary antibodies (PCNA, cyclin D1, CDK4, caspase‐8, caspase‐9, caspase‐3, VEGF, MMP‐2 and MMP‐9 at dilution of 1:250; Smad2/3, p‐Smad2/3, E‐cadherin, vimentin and β‐actin at dilution of 1:1000) overnight at 4°C. After washing three times with 0.5% tween‐20 in tris‐buffered saline (TBS), the membranes were incubated with a 1:1000 dilution of the relevant biotin‐conjugated secondary antibodies (Santa Cruz, CA, USA) for 2 h at 25°C, followed by incubation with peroxidase‐conjugated streptavidin (Jackson ImmunoResearch Inc.) for 1 h. Finally, the membranes were exposed to enhanced chemiluminescence reagent (ECL, T‐Pro Biotechnology) and detected using a fluorescence/chemiluminescence imaging analyser (GE LAS‐4000, GE healthcare Life Sciences). The ImageJ 1.47t software was used for densitometry analysis and normalized against β‐actin expression.

### 2.7 Wound‐healing assay

B16F10 cells were harvested and 1 × 10^6^ cells were seeded into a 6‐well plate and incubated for 24 h. When cells reached 90% confluence, the cell monolayer was scratched with the tip of a P200 pipette to create a straight wound. After washing with PBS, cells were treated with IC_20_, IC_40_ and IC_60_ concentrations of PA (0, 22.5, 45.0 and 67.5 μM) in the medium with 1% FBS, and then observed and imaged at 0, 24 and 48 h under an inverted microscope. ImageJ 1.47 software was used to measure the space of wound closure, and the migration area (%) was calculated based on the negative control.

### 2.8 In vivo experiments in mice

The anti‐tumour activity of PA in vivo was studied according to the prescribed guidelines for the Care and Use of Laboratory Animals. The animal experimental study was performed at Chung Shan Medical University (CSMU) and was approved by the Institutional Animal Care and Use Committee (IACUC) of CSMU (allowance number: CSMU‐IACUC‐2033). The 6–8‐week‐old C57/BL6 mice were purchased from the National Laboratory Animal Center and were adapted to laboratory conditions for a week. B16F10 cells (1 × 10^6^ cells in 100 μL PBS) were subcutaneously injected into the right hind flank of the mice. After 5 days, the mice were randomly divided into four groups and treated with vehicle (mineral oil), 75 mg/kg PA or 150 mg/kg PA via subcutaneous injection every 2 days. Cisplatin was administered by intraperitoneal route once a week at the dose of 2.5 mg/kg for 3 weeks as a positive control. Tumour volume (length × width × depth × π/6 mm^3^) and body weight were recorded thrice a week. Mice were sacrificed using carbon dioxide when the tumour volume exceeded 1500 mm^3^. Tumour tissues were fixed in 10% formalin, embedded in paraffin and sliced, followed by staining with TUNEL, haematoxylin and eosin (H&E) and IHC (caspase‐3, PCNA, VEGF, MMP‐2 and MMP‐9) for further histological examination.^28^

### 2.9 Colony formation assay

A plate clonogenic assay was used to test the colony formation ability of B16F10 cells. The cells were digested and counted, and approximately 2000 cells were seeded into a 24‐well plate and incubated for 24 h. The cells were treated with 45.0 μM PA and/or 6.6 μM cisplatin in triplicate for 24 h, followed by culturing in a drug‐free medium with 5% FBS for another 7 days. Subsequently, the cells were washed with PBS, fixed with 100% methanol and stained with 0.1% crystal violet and then colonies (>50 cells) in each well were counted using a light microscope.

### 2.10 Migration assay

The Boyden chamber assay was used to detect the migration of melanoma cells. Briefly, B16F10 cells were starved for 24 h, 1.0 × 10^5^ cells suspended in 100 μL serum‐free medium were added to the upper chamber, and 100 μL culture medium (10% FBS) containing 67.5 μM PA and/or 6.6 μM cisplatin was added to the lower chamber. After incubation at 37° for 24 h, the cells in the upper chambers were removed, then the migrated cells were fixed with 100% methanol and stained with 0.1% crystal violet for 10 min each. Images of migrated cells in five randomly selected fields were captured, and cell numbers were calculated under a light microscope.

### 2.11 In vitro resistance assay

B16F10 cells were plated in 96‐well plates and allowed to attach for 24 h. PA (89.9 μM) and/or cisplatin (6.6 μM) were added in three technical replicates for 5 and 10 days, and fresh media and drugs were added every 3 days for the duration of the experiment. The treated cells were fixed with 100% methanol and stained with 0.1% crystal violet. Finally, 50 μL of 10% acetic acid was added to each well and the OD was measured at 560 nm using a microplate reader. Cell survival (%) = (PA − treated OD/control OD on day 5) × 100.

### 2.12 Statistical analysis

Data are presented as the mean ± SD (in vitro) or mean ± SEM (in vivo). The unpaired Student's *t*‐test was used to compare two groups, one‐way anova was used to compare multiple groups and the Kaplan–Meier method was used to perform survival analysis, using Excel 2016 software or SPSS v16.0 software. Statistical significance was set at *p* < 0.05.

## 3 RESULTS

### 3.1 PA inhibited cell growth and induced cell cycle arrest in G_0_ /G_1_ phase

**FIGURE 1:**
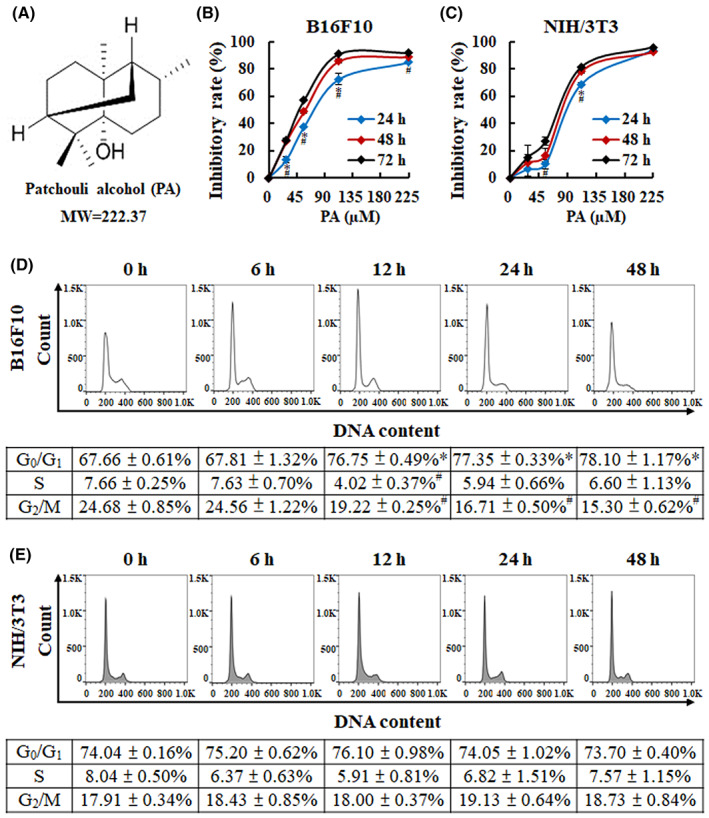
The effects of PA on cell growth and cell cycle distribution of B16F10 cells. (A) Chemical structure illustration of PA from the database of ChemFaces. (B, C) Melanoma cells (B16F10) and normal fibroblasts (NIH/3T3) were treated with PA (0–224.9 μM) for 24, 48 and 72 h, and cell viability was detected by an MTT assay. *P < 0.05 versus 48 h. ^#^ *P* < 0.05, versus 72 h. Effects of PA on cell cycle distribution in B10F10 melanoma cells (D) and NIH/3T3 fibroblast (E) treated with PA (89.9 μM) for 0–48 h were determined by flow cytometry analysis. Results are presented as the mean ± SD of at least three independent experiments. *P < 0.05, versus control with a significant increase. ^#^ *P* < 0.05, versus control with significant decrease.

The chemical structure of PA with a molecular weight of 222.37 g/mol is shown in Figure 1A. First, to investigate whether PA exerted inhibitory effects on melanoma cells, MTT assays were performed to determine the inhibition rate and calculated the IC_50_ concentration. Melanoma cells were treated with serially diluted PA for 24, 48 and 72 h. Compared to NIH/3T3 normal fibroblast cells, PA exhibited higher inhibitory effects on the proliferation of B16F10 melanoma cells in a time‐ and dose‐dependent manner (Figure 1B,C). The IC_50_ values of PA in B16F10 cells at 48 h were 58.5 ± 2.2 μM, which was lower than that in NIH/3 T3 cells (86.8 ± 13.5 μM); however, IC_50_ values of cisplatin in B16F10 cells (19.3 ± 0.3 μM) was higher than that in NIH/3 T3 cells (11.0 ± 1.0 μM). The results indicated that PA selectively inhibited the growth of melanoma cells, whereas cisplatin had no such effect. We then evaluated the effect of PA on cell cycle distribution using flow cytometry analysis in B16F10 and NIH/3T3 cells. The distribution of PA‐treated cells in the G_0_/G_1_, S and G2/M phases was calculated using FlowJo software. In B16F10 cells, the percentage of cells in the G_0_/G_1_ phase was significantly increased, while the percentage of cells in the G2/M phase was significantly decreased by treatment with PA, compared with the control group (Figure 1D), suggesting that PA was able to induce cell cycle arrest in the G_0_/G_1_ phase in melanoma cells. However, there was no significant change in NIH/3T3 cells after PA treatment within 48 h (Figure 1E). Above all, these results demonstrated that PA could inhibit the proliferation of melanoma cells via the induction of cell cycle arrest, which may be a potential drug for melanoma treatment.

### 3.2 PA influenced the cell morphology and triggered apoptosis in melanoma cells

**FIGURE 2:**
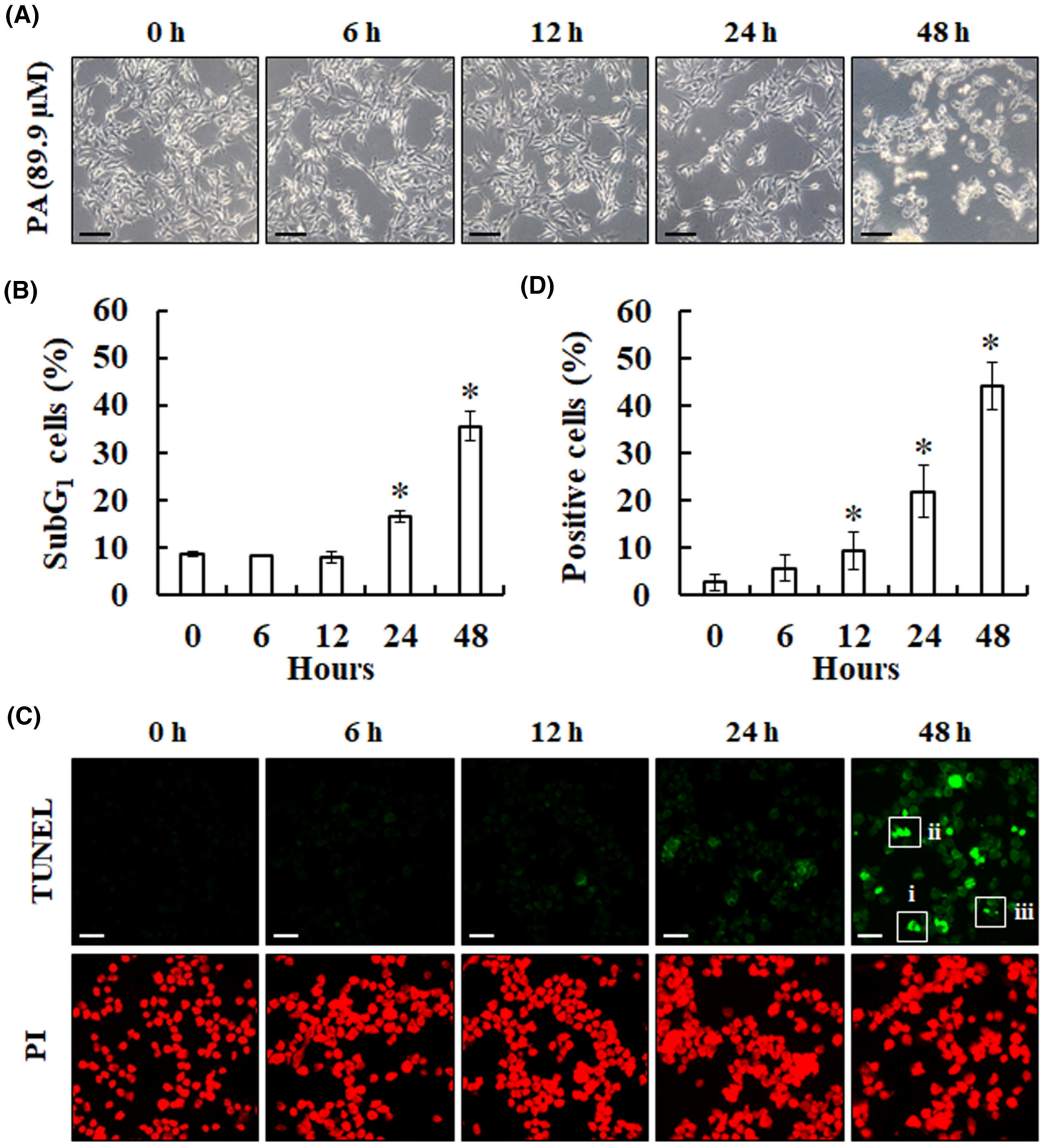
The cells treated with PA showed apoptotic morphologies and characteristics. (A, B) After stimulation with 89.9 μM PA for 0–48 h, B16F10 cells were observed for cell morphology under an inverted microscope (scale bar: 100 μm) and the percentage of subG_1_ phase was detected by flow cytometry analysis. Data are represented as means ± SD. *P < 0.05 versus control. (C, D) To determine apoptosis, B16F10 cells were treated with 89.9 μM PA for 0–48 h, stained with TUNEL and observed using the ZEISS Axioskop 2 microscope with a FITC filter, 400× magnification. Scale bar: 50 μm. It indicated chromatin condensation (i), DNA fragmentation (ii) and apoptotic bodies (iii). PI (red fluorescence) was used as the contrast dye to highlight the nucleus.

As shown in Figure 2A, the morphology of B16F10 cells exhibited significant changes after treatment with PA. The PA‐treated cells began to shrink, turned circular, the cell edges became irregular and many dead cells were suspended in the medium. Besides, the percentage of cells at subG_1_ phase was enhanced from 8.54% ± 0.56% to 8.32% ± 0.31%, 7.95% ± 1.27%, 16.54% ± 1.30% and 35.58 ± 3.18%, respectively, with increasing PA treatment time (Figure 2B). To determine cells undergoing apoptosis, a TUNEL assay was performed to evaluate the pro‐apoptotic effect of PA on B16F10 cells. Cells treated with 89.9 μM PA for 48 h showed many fragmented nuclei with green fluorescence and apoptotic morphology, such as chromatin condensation, DNA fragmentation and apoptotic bodies (Figure 2C). The number of TUNEL‐positive cells was enhanced with increasing PA treatment time, measured by cell counting (Figure 2D). These results demonstrated that PA exerted time‐dependent pro‐apoptotic effects on B16F10 cells.

### 3.3 PA decreased expression of cyclin D1/CDK4 and enhanced activation of caspases

**FIGURE 3:**
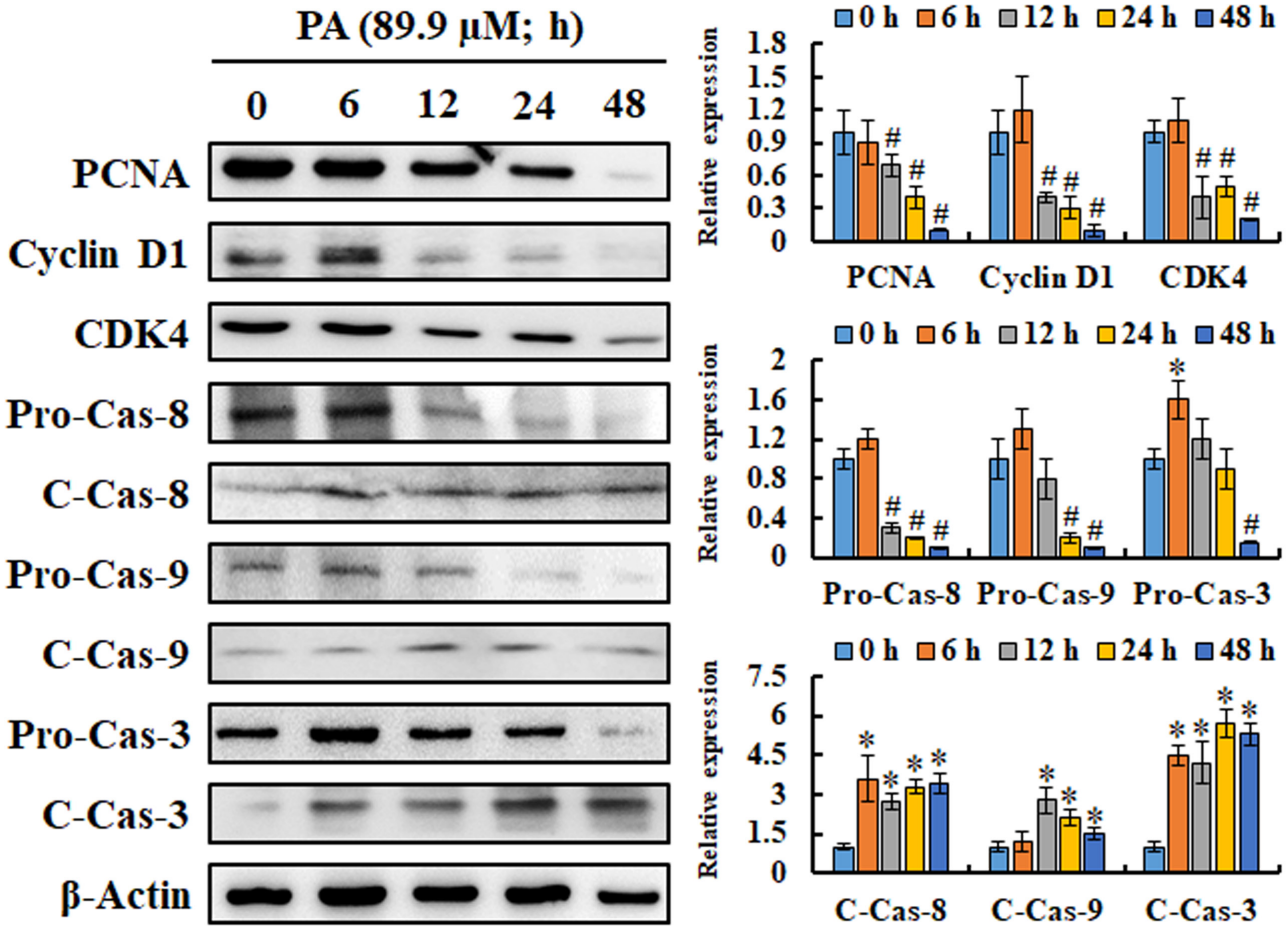
The expression levels of cell cycle and apoptosis relative proteins regulated by PA treatment. Cells were incubated with 89.9 μM of PA for 0, 6, 12, 24 and 48 h, and the expression level of proteins associated with cell cycle and apoptosis was detected using western blotting. PA treatment dramatically attenuated the expressions of PCNA, cyclin D1 and CDK4, while activating caspase‐8, caspase‐9 and caspase‐3. Data are represented as means ± SD of at least three independent experiments. *P < 0.05, versus control with a significant increase. ^#^ *P* < 0.05, versus control with a significant decrease. C‐Cas, cleaved‐caspase; PCNA, proliferating cell nuclear antigen; Pro‐Cas, pro‐caspase.

Based on the special phenomenon of cell cycle arrest and apoptosis induction, we further measured the effects of PA on the expression of the relevant proteins by western blotting. Immunoblot results indicated that the expression of proliferating cell nuclear antigen (PCNA) was decreased by PA treatment (Figure 3), and the reduced expression of cyclin D1 and CDK4, as representative markers of the cell cycle at the G_0_/G_1_ phase, matched the above results of the cell cycle. Furthermore, the expression of pro‐caspase‐8, 9 and 3 was significantly reduced, while cleaved‐caspase‐8, 9 and 3 was significantly increased with treatment time, suggesting that extrinsic and intrinsic apoptosis pathways were activated by PA treatment in melanoma cells.

### 3.4 PA attenuated the migratory abilities of B16F10 cells

**FIGURE 4:**
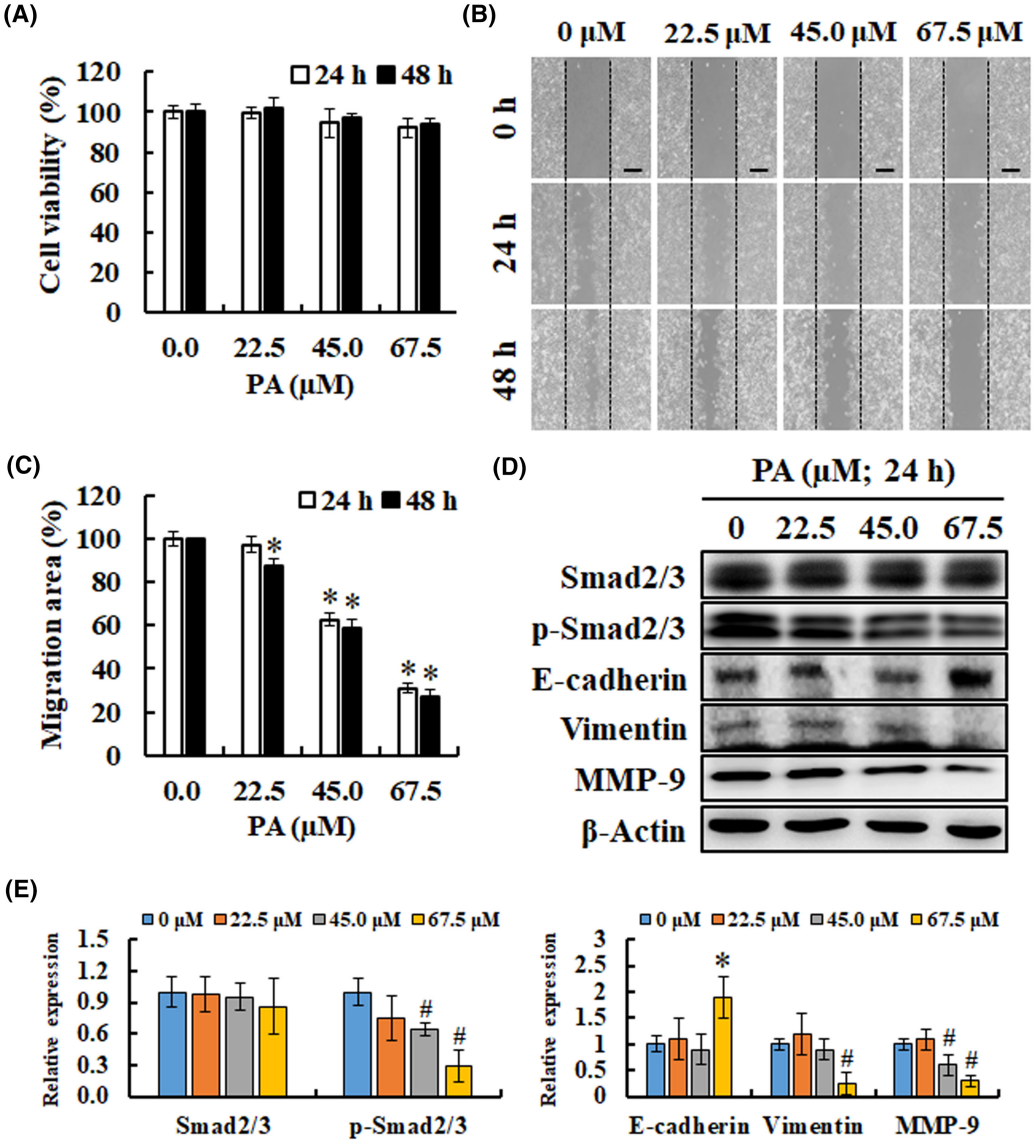
The effects of PA on cell migration in B16F10 melanoma cells. (A) The inhibitory effects of PA (0, 22.5, 45.0 and 67.5 μM) in a medium containing 1% FBS on cells at 90% confluence. (B, C) Wound closure analysis for B16F10 cells was explored at 24 and 48 h, and the histograms of the percentage of wound closure were calculated by ImageJ 1.47 software. Treatment with PA remarkably inhibited the migratory ability of the B16F10 cells in a wound‐healing assay. Scale bar: 200 μm. Data are shown as means ± SD. *P < 0.05, versus control. (D, E) The protein expressions of epithelial‐mesenchymal transition and tumour invasion markers were determined by western blotting after PA treatment with β‐Actin as the internal control. *P < 0.05, versus control with a significant increase. ^#^ *P* < 0.05, versus control with a significant decrease. MMP, matrix metalloproteinases.

The metastatic ability of tumour cells poses a major threat to cancer‐related mortality; thus, the present study investigated whether PA affects the migration capability of melanoma cells. First, there was no significantly inhibitory effect for PA (0, 22.5, 45.0 and 67.5 μM) in a medium containing 1% FBS when cells reached 90% confluence (Figure 4A). Then wound‐healing assay was used to verify the anti‐migration effect of PA on B16F10 cells. After cells treated with IC_20_, IC_40_ and IC_60_ concentrations of PA (0, 22.5, 45.0 and 67.5 μM) for 48 h, the percentages of migration area were significantly reduced from 100% ± 4.63% to 87.65% ± 2.94%, 58.94% ± 3.87% and 27.18% ± 3.11%, respectively, suggesting that PA inhibited the migration of B16F10 cells in a dose‐dependent manner (Figure 4B,C). In addition, the western blotting results indicated that PA treatment triggered an increase in E‐cadherin expression and a decrease in p‐Smad2/3, vimentin and MMP‐9 expression (Figure 4D,E). These results demonstrate that PA inhibited the motility of B16F10 cells in vitro.

### 3.5 PA suppressed tumour growth of melanoma in vivo

**FIGURE 5:**
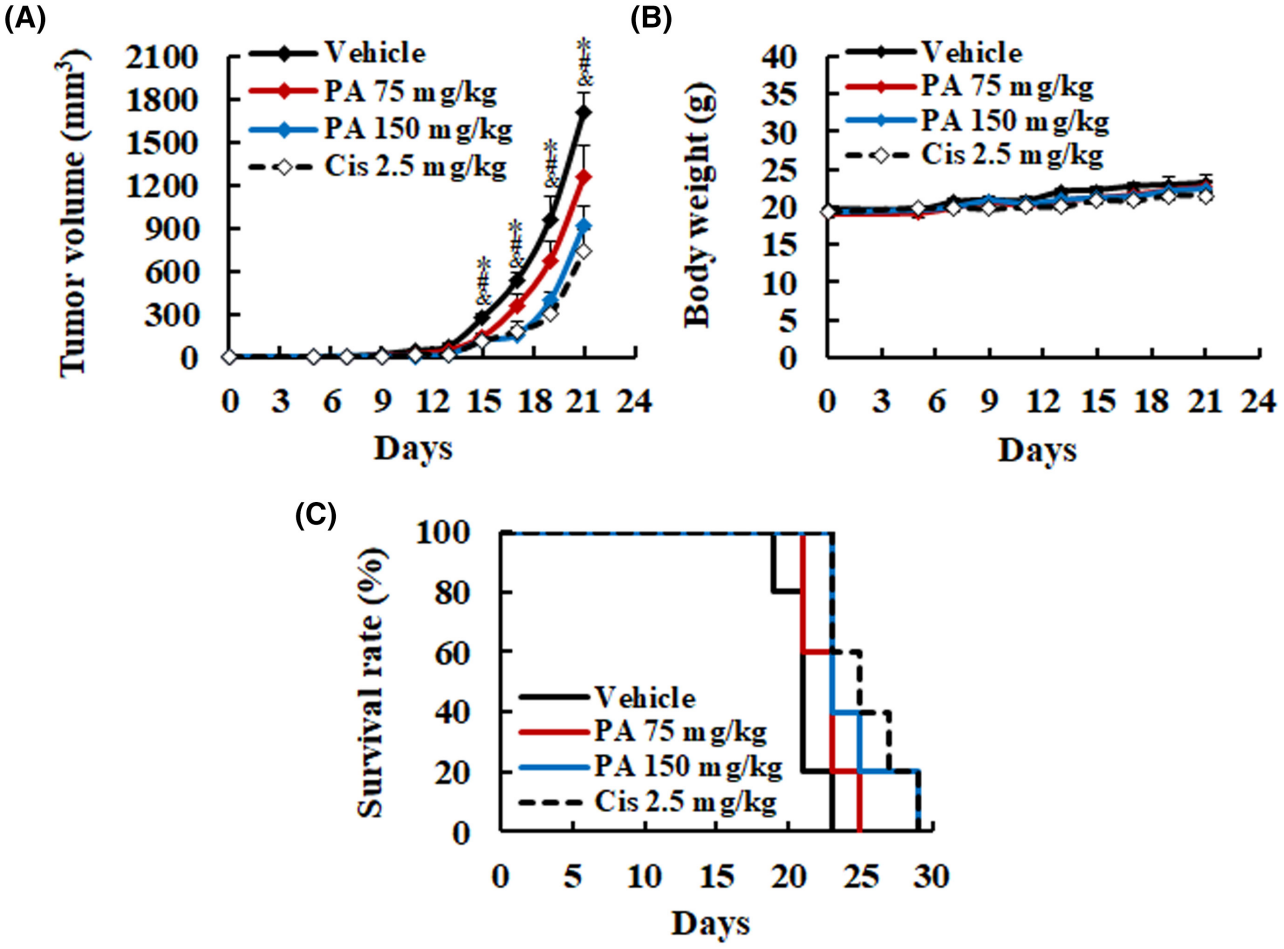
Suppressive effects of PA against melanoma in subcutaneous animal model. Mice bearing B16F10 melanoma cells were subcutaneously treated with vehicle (mineral oil, *n* = 5), 75 mg/kg PA (*n* = 5) or 150 mg/kg PA (*n* = 5) once per 2 days. Cisplatin (2.5 mg/kg, *n* = 5) was administered by intraperitoneal route once a week for 3 weeks. PA treatment significantly inhibited tumour growth (A) and increased survival rate (B), analysed using the unpaired Student's *t*‐test or Kaplan–Meier method (*P* < 0.05), respectively. (C) Body weights from vehicle or treated groups of mice were obtained every 2 days and revealed that PA had no effect on the body weight of mouse. Data are presented as mean ± SEM. *P < 0.05, versus 75 mg/kg PA. ^#^ *P* < 0.05, versus 150 mg/kg PA. ^&^ *P* < 0.05, versus Cisplatin. Cis, Cisplatin.

To further determine the functions of PA, tumorigenicity analysis was conducted in C57/BL6 mice injected with B16F10 cells and treated with PA. The 75 and 150 mg/kg of PA treatment significantly reduced tumour size (1262.55 ± 217.24 mm^3^; 918.60 ± 128.75 mm^3^) compared with the vehicle group (1701.30 ± 139.87 mm^3^) at day 21 (Figure 5A), and prolonged life span from 23 days to 25 and 29 days (Figure 5B), respectively. However, there was no significant difference in the weight of mice between the vehicle and PA groups (Figure 5C). Although cisplatin group has the most obvious effects on inhibiting tumour growth, the body weight of mice is slightly lower than other groups, indicating that it may have more toxicity during treatment.

**FIGURE 6:**
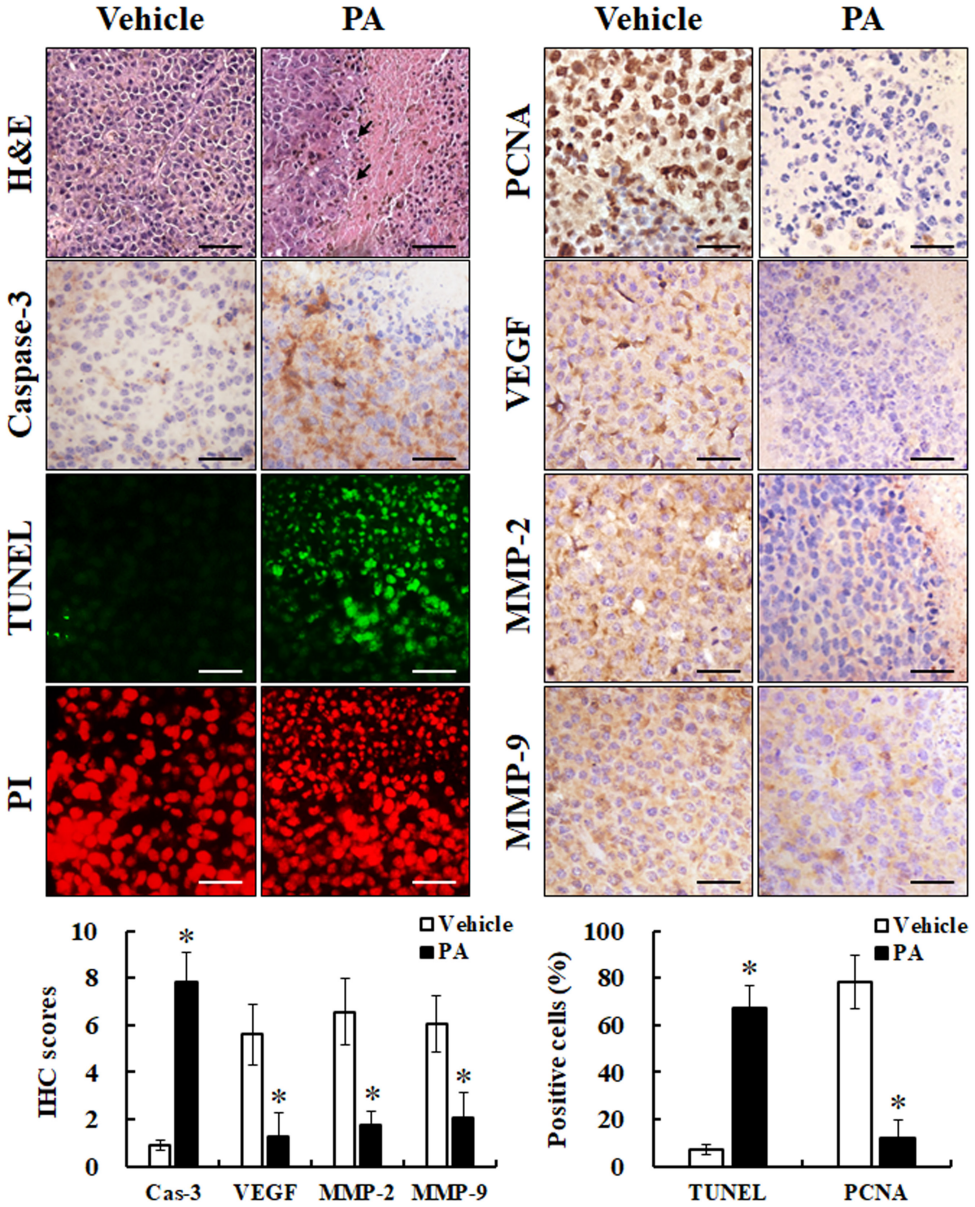
PA regulated markers expression of proliferation, apoptosis, angiogenesis and metastasis in vivo. The mice (vehicle and 150 mg/kg PA groups) were sacrificed when the tumour volume exceeded 1500 mm^3^, and then the tumour was fixed, sliced and stained with H&E (scale bar: 100 μm), IHC (scale bar: 50 μm) and TUNEL (scale bar: 50 μm) for histological examination. Image of H&E staining showed the morphology of cell death and nucleolysis (arrow). The expression of caspase‐3, PCNA, VEGF, MMP‐2 and MMP‐9 were examined using immunohistochemical analysis and scored using the Quickscore method. PA‐induced cell apoptosis was determined using tissue TUNEL stain. All data are shown as mean values ± SEM. *P < 0.05, versus vehicle group. H&E, haematoxylin and eosin; IHC, immunohistochemistry; MMP, matrix metalloproteinases; PCNA, proliferating cell nuclear antigen; TUNEL, terminal deoxynucleotidyl transferase dUTP nick end labelling; VEGF, vascular endothelial growth factor.

To study the underlying mechanism of PA in inhibiting melanoma, histological analysis was used to detect markers of apoptosis, proliferation, angiogenesis and metastasis in vivo. As shown in Figure 6, PA treatment induced cell death and nucleolysis, enhanced the expression of activated caspase‐3 and increased the number of apoptotic cells, as determined by TUNEL staining. Meanwhile, the expression of PCNA, VEGF, MMP‐2 and MMP‐9 was dramatically reduced in tumour‐bearing mice treated with PA in comparison with the vehicle group. Taken together, these results indicate that PA suppresses the growth of melanoma in vivo via inhibition of proliferation and induction of apoptosis, which is in accordance with the in vitro experiments.

### 3.6 PA combined with cisplatin reduced colony formation, migration and drug resistance abilities

**FIGURE 7:**
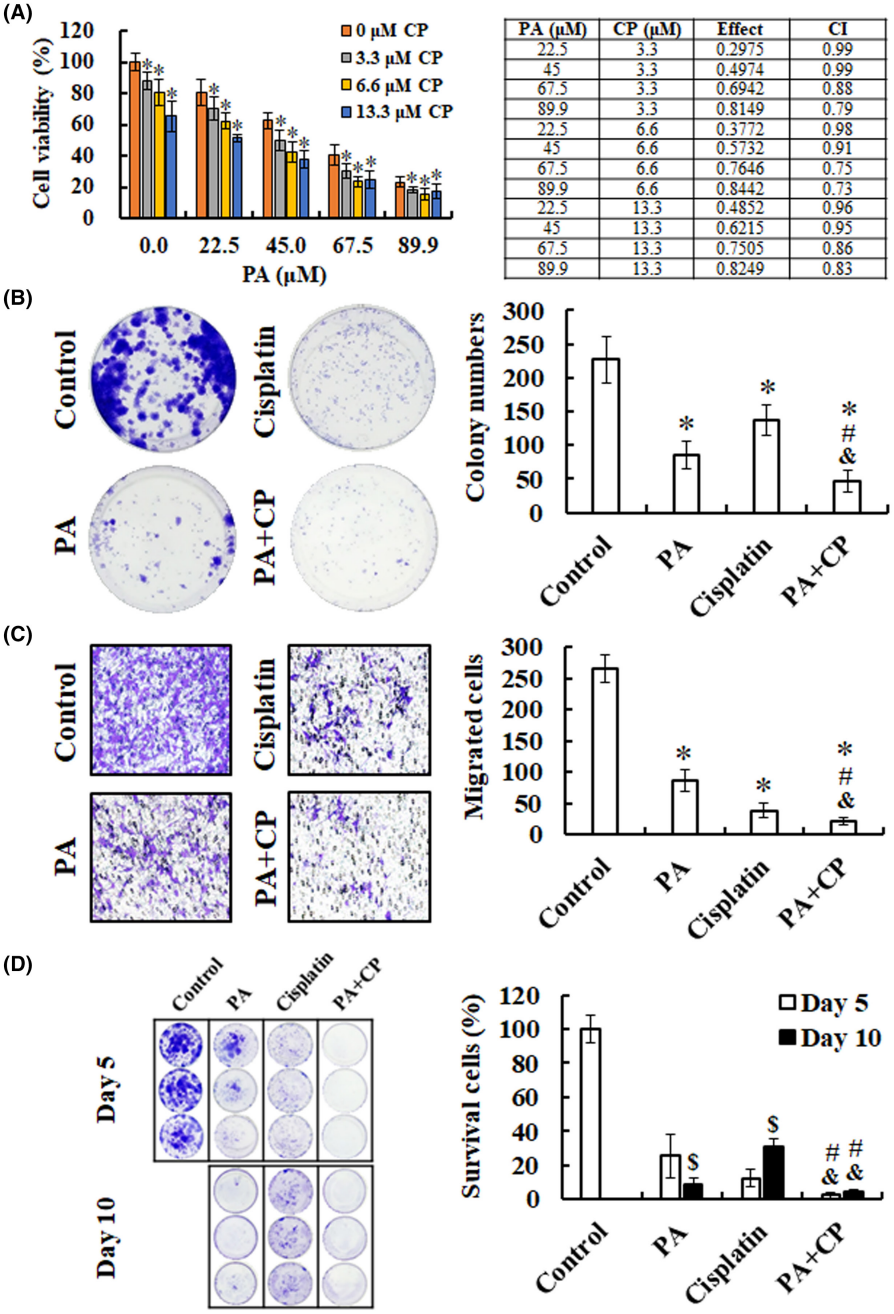
Combination effects of PA combined with cisplatin on proliferation, colony formation, migration and drug‐resistant abilities. (A) Cells were treated with indicated concentrations of PA and cisplatin for 48 h, and measured cell viability using MTT assay. The combination index (CI) was calculated by CompuSyn software, using to evaluate the synergism (CI < 1), additive effects (CI = 1) and antagonism (CI > 1). *P < 0.05, versus PA only. (B) B16F10 cells were treated with PA (45.0 μM) and/or cisplatin (6.6 μM) for 24 h and subsequently cultured in drug‐free medium containing 5% FBS for another 7 days. The colonies were fixed using 100% methanol, stained with crystal violet and counted microscopically. (C) Boyden chamber assay of B16F10 cells upon treatment with PA (67.5 μM) and/or cisplatin (6.6 μM) for 24 h, after which the migrated cells were stained, photographed and quantified. (D) For in vitro resistance assay, after long‐term culture with PA (89.9 μM) and/or cisplatin (6.6 μM) for 5 and 10 days, the treated cells were fixed, stained and dissolved in 10% acetic acid, followed by O.D. measurement at 560 nm using a microplate reader. Survival cells of the control group at day 5 were considered as 100%. Data are represented as means ± SD in three technical replicates. *P < 0.05, versus control. ^#^ *P* < 0.05, versus PA. ^&^ *P* < 0.05, versus cisplatin. ^$^ *P* < 0.05, versus day 5. CP, cisplatin.

To investigate the effects of PA combined with cisplatin, MTT assay, colony formation, cell migration and in vitro resistance assays were performed on B16F10 cells treated with the drug combination. The results showed that PA combined with cisplatin significantly reduced cell viabilities compared with PA or cisplatin alone, and all the CI values were <1, indicating the drug combination had synergistically inhibitory effects in melanoma (Figure 7A). As shown in Figure 7B, the combination of PA and cisplatin significantly decreased colony numbers (46.2 ± 15.9) compared with PA (84.5 ± 20.4) or cisplatin (136.4 ± 22.6) alone. Moreover, the results of the Boyden chamber assay demonstrated that the numbers of cells treated with drug combination (22.2 ± 5.8 cells) that migrated to the lower surfaces of the membranes were less than each drug alone (PA, 86.7 ± 17.1 cells; cisplatin, 38.6 ± 11.9 cells, Figure 7C). To determine the improvement of cisplatin resistance by combination treatment with PA, B16F10 cells were long‐term cultured in a medium with cisplatin and/or PA. The treated cells were inhibited at day 5 and re‐grew at day 10, indicating that the cells developed resistance to cisplatin; however, the combination of drugs reduced and delayed this effect (Figure 7D). Taken together, these results demonstrate that PA enhances the anti‐proliferative and anti‐migratory activity of cisplatin and attenuates the development of cisplatin resistance in melanoma cells.

## 4 DISCUSSION

Compounds from natural sources have been widely investigated for cancer therapy because of their availability and low toxicity compared to standard chemotherapeutic agents.^11^ *Pogostemon cablin* Benth. is an important medicinal plant species, a source of bioactive compounds, and has shown inhibitory activity against colorectal cancer, hepatocellular carcinoma and myeloid leukaemia.^29^, ^30^, ^31^ Its activity can be attributed to PA, which is one of the active compounds responsible for its inhibitory effects on cancer cells. A previous study indicated that PA showed anti‐inflammatory activity by downregulating the extracellular signal‐regulated kinase (ERK)‐mediated nuclear factor kappa B (NF‐κB) pathway in tumour necrosis factor (TNF)‐α‐stimulated HT‐29 human colorectal cancer cells.^20^ Growth inhibition and apoptosis were observed in PA‐treated HCT116 and SW480 colorectal cancer cells, induced by a decrease in histone deacetylase 2 (HDAC2) expression and HDAC enzyme activity, and subsequent repression of c‐Myc and activation of the NF‐κB pathway.^25^ In addition, PA induced cell cycle arrest and apoptosis in vitro and in vivo by blocking phosphorylation of the epidermal growth factor receptor (EGFR) pathway and activation of the c‐Jun N‐terminal kinase (JNK) signalling pathway in A549 human non‐small cell lung cancer cells.^26^ It also inhibited cell proliferation, migration and invasion, as well as induced apoptosis by regulating the mitogen‐activated protein kinase (MAPK) and PI3K/AKT signalling pathways in NCI‐N87 and HGC‐27 human gastric cancer cells.^32^ The anti‐cancer effects of PA have been previously studied in gastrointestinal and lung cancers; however, this study focused on examining the effect of PA on B16F10 melanoma cells. The results suggested that PA significantly inhibited the growth of B16F10 cells by inducing cell cycle arrest and apoptosis. The migratory ability of B16F10 cells was attenuated after PA treatment in a dose‐dependent manner. In animal studies, PA suppressed tumour progression by regulating proliferation, apoptosis, angiogenesis and invasion‐related protein expression. Moreover, the combination of PA and cisplatin synergistically inhibited colony formation, migration and drug resistance development. Our findings indicate that PA may have potential uses in the development of nutritional supplements and pharmaceuticals for the prevention and treatment of melanoma.

At present, an increasing number of researchers believe that inducing cell cycle arrest and apoptosis may be a new therapeutic strategy for cancer prevention and treatment. The cell cycle is strictly regulated by these genes. The key to carcinogenesis is uncontrolled cell growth and proliferation due to abnormal regulation of the cell cycle.^33^ Based on changes in DNA content, the cell cycle is divided into four stages: G_1_, S, G2 and M phases. In the current study, the cell cycle was analysed using PI staining and flow cytometry to identify the effects of PA treatment on the cell cycle. The results revealed that the cell cycle of B16F10 cells exposed to PA was arrested in the G_0_/G_1_ phase. A combination of cyclins, CDKs, and CDK inhibitors mediates the regulation of cell cycle distribution at critical restriction points.^34^ Among them, cyclin D1 and CDK4 are important regulators that promote the transition from G_1_ to S phase. We detected the expression of proteins associated with cell cycle arrest through western blotting and found that the expression of cyclin D1 and CDK4 decreased in a time‐dependent manner after PA treatment.

It is well known that the induction of cell cycle arrest can trigger apoptosis. Apoptosis is an essential mechanism that induces cell death triggered by multiple signalling pathways and is characterized by the shrinkage of the cell and nucleus, chromatin concentration, DNA fragmentation and the formation of apoptotic bodies.^35^ In the present study, PA‐treated cells showed an increasing percentage of cells in the subG_1_ phase and typical apoptotic cell morphology, indicating that PA caused cell death of melanoma cells via induction of apoptosis. Studies have shown that apoptosis is mainly mediated through the death receptor‐mediated (extrinsic) or mitochondrial (intrinsic) pathways. The complex cascade of caspases plays an important role in the process of apoptosis, in which the activation of caspase‐8 and caspase‐9 (initiator caspases) is characteristic of the extrinsic and intrinsic pathways, respectively.^36^ Thus, to investigate the pathway of apoptosis induced by PA, the activation of caspase‐3, an important effector caspase, caspase‐8 and caspase‐9 was analysed by western blotting. The results showed that treatment of melanoma cells with PA resulted in cleavage/activation of caspase‐8, caspase‐9 and caspase‐3, suggesting that PA induced apoptosis via activation of both extrinsic and intrinsic pathways.

The hallmark migratory and invasive ability is the primary cause of high mortality in melanoma, and it goes through a complex process regulated by a series of molecular mechanisms.^37^ Thus, inhibiting cancer cell migration is an important strategy for melanoma treatment. The present study used wound‐healing and Boyden chamber assays to evaluate the effect of PA on the migration ability of B16F10 cells. The results showed a significant reduction in the migration of melanoma cells following PA treatment. Epithelial‐mesenchymal transition (EMT) is a process in which cells lose epithelial characteristics and obtain mesenchymal characteristics and is related to the various tumour functions, such as tumorigenesis, malignant progression, migration, metastasis and drug resistance.^38^ TGF‐β‐induced activation of the receptor complex results in the phosphorylation of Smad2 and Smad3, which then form trimers with Smad4, translocate into the nucleus and cooperate with DNA‐binding transcription factors to repress or activate the transcription of target genes, such as E‐cadherin and vimentin.^39^ EMT is usually defined as the downregulation of the epithelial marker E‐cadherin and upregulation of the mesenchymal marker vimentin.^40^ MMP are zinc‐dependent endopeptidases that play key roles in the migration and invasion of cancer cells through the degradation of the extracellular matrix. Among the members of the MMP family, MMP‐2 and MMP‐9 have been reported to correlate with the invasive phenotype of melanoma.^41^ Therefore, we investigated the effects of PA on the expression of migration‐associated proteins in melanoma cells. This study demonstrated that PA inhibits the migratory ability of B16F10 cells by inhibiting phosphorylation of Smad2 and Smad3 leading to upregulation of E‐cadherin and downregulation of vimentin, MMP2 and MMP9.

As a well‐known chemotherapeutic drug, cisplatin has been widely used to treat a variety of solid cancers, including ovarian, lung and testicular cancer.^42^ The most important anticancer mechanism of cisplatin is to trigger DNA damage and induce cell apoptosis, leading to the death of cancer cells. Cisplatin is beneficial for the overall survival and relapse‐free survival of patients with advanced melanoma and metastatic melanoma.^43^ However, single‐agent chemotherapy has low response rates with a median duration of approximately 3 months and undesirable side effects such as ototoxicity, nephrotoxicity, hepatotoxicity and gastrointestinal toxicity.^42^, ^44^ To overcome this limitation, combinations of cisplatin with other agents or natural products with different mechanisms of action have been widely reported.^42^, ^45^ The current study showed that PA combined with cisplatin exhibited synergistic inhibitory effects on the colony formation and migration of B16F10 cells. In addition, PA strongly reduced the regrowth of cisplatin‐treated cells, indicating that PA attenuated the development of resistance to cisplatin. Taken together, PA may not only be a potential approach for melanoma treatment but also a sensitizer for cells resistant to cisplatin or other conventional chemotherapeutic drugs, suggesting that it has good clinical application prospects.

Because human melanoma is an aggressive cancer type, in vitro and in vitro experiments were performed in the current study using the highly aggressive murine melanoma cell line B16F10 to evaluate the inhibitory effect of PA on melanoma cells and animal models, respectively. B16 melanoma, a spontaneous melanoma derived from C57BL/6 mice, is the most commonly used model of metastatic melanoma for preclinical studies. The B16F10 cell line, the most frequently used variant, was generated as the 10th serial passage subclone of B16 parent cells, as well as has highly aggressive ability, which will metastasize from the primary subcutaneous site to the lung in animal model.^46^ The genetics and histological characteristics of murine melanoma are known to be similar to those of human melanoma.^47^ PA exhibited growth inhibitory activity not only against mouse melanoma B16F10 cells, but also against human prostate cancer cells (PC3 and DU145), lung cancer cells (A549) and colorectal cancer cells (HCT116 and SW480),^25^, ^26^, ^27^ suggesting that it had the potential of anti‐cancer in both mouse and human cancers. The findings indicate that PA may be a potential compound for the development of novel drugs or adjuvants that can be used in combination with clinical drugs to improve side effects and drug resistance. More studies are needed, preferably using primary human melanoma, are needed to determine the potential and clinical application of PA in the therapy of metastatic melanoma.

In conclusion, this study provides evidence that PA exhibits potent inhibitory activity against melanoma cell proliferation in vitro and in vivo. PA treatment effectively induced cell cycle arrest at the G_0_/G_1_ phase via downregulation of cyclin D1/CDK4 and triggered apoptosis via the activation of extrinsic and intrinsic pathways. It reduces the migratory ability of melanoma cells through the regulation of E‐cadherin/vimentin and MMP‐2/MMP‐9 expression. In addition, the combination of PA and cisplatin showed synergistic inhibitory effects on colony formation and migration, and attenuated the development of drug resistance. These findings suggest that PA may be a potential anticancer candidate for the treatment of melanoma.

## AUTHOR CONTRIBUTIONS

**Kai‐Fu Chang:** Conceptualization (equal); data curation (equal); formal analysis (equal); investigation (equal); writing – original draft (equal). **Hung‐Chih Lai:** Data curation (equal); formal analysis (equal); investigation (equal); methodology (equal); writing – original draft (equal). **Shan‐Chih Lee:** Data curation (equal); formal analysis (equal); methodology (equal); software (equal). **Xiao‐Fan Huang:** Conceptualization (equal); data curation (equal); formal analysis (equal). **Ya‐Chih Huang:** Methodology (equal); software (equal). **Tien‐Erh Chou:** Data curation (equal); formal analysis (equal). **Chih‐Yen Hsiao:** Conceptualization (equal); funding acquisition (equal); project administration (equal); writing – review and editing (equal). **Nu‐Man Tsai:** Conceptualization (equal); funding acquisition (equal); project administration (equal); supervision (lead); writing – review and editing (equal).

## FUNDING INFORMATION

This research was funded by Ditmanson Medical Foundation Chia‐Yi Christian Hospital (grant number R110‐01), Shin‐Kong Wu Ho‐Su Memorial Hospital (grant number 2020SKHADR009 and 2021SKHADR006) and the Ministry of Science and Technology, Taiwan (grant number MOST 109‐2320‐B‐040‐012, MOST 110‐2320‐B‐040‐006 and MOST 111‐2320‐B‐040‐022).

## CONFLICT OF INTEREST STATEMENT

The authors declare no conflict of interest.

## Data Availability

The data presented in this study are available from the corresponding author upon reasonable request.
